# Incidence of Health Problems in Australian Mixed Martial Arts and Muay Thai Competitors: A 14-Month Study of 26 Combat Sports Events

**DOI:** 10.1186/s40798-025-00880-3

**Published:** 2025-05-28

**Authors:** Colin S. Doherty, Oliver R. Barley, Lauren V. Fortington

**Affiliations:** School of Medical and Health Sciences, Edith Cowan University, 270 Joondalup Drive, Joondalup, WA 6027 Australia

## Abstract

**Background:**

Mixed martial arts (MMA) and Muay Thai (MT) are widely practiced combat sports, yet research on the full spectrum of competition-related health problems (HPs) remains limited, particularly for MT. Existing studies in both sports primarily focus on retrospective analyses of severe injuries, often estimating time lost from training or competition. This study describes the incidence of all competition HPs reported seven days after MMA and MT contests, and determines the number of days impacted by tracking athletes’ self-identified worst HPs until resolution.

**Methods:**

Data on competition HPs were collected using an online questionnaire completed seven days after each MMA and MT event (*n* = 26). The questionnaire included the Oslo Sports Trauma Research Centre Questionnaire on Health Problems 2 (OSTRC-H2). The Combat Sports Commission of Western Australia provided competition exposure time data. Incidence rates of HPs were calculated per 100 min of exposure (HPIR_ME_). Competitors reporting HPs were followed up weekly using the OSTRC-H2 questionnaire until their worst HPs resolved.

**Results:**

Of the 175 competitors (238 responses) who completed the questionnaire (76% male; age: 27 ± 6 years), 81 competitors (92 responses) reported a total of 411 HPs (315 injuries, 96 illnesses). Among the 92 worst HPs, 26 were substantial, and 24 prevented training. The HPIR_ME_ was 20.1 (95% CI: 16.5–24.4) for MMA and 25 (95% CI: 22.3–28) for MT. Follow-up captured 78 (85%) of the worst HPs, with 175 responses collected over 14–70 days post-competition. The median days impacted by the worst HPs were 20 for MMA and 16 for MT.

**Conclusions:**

Among respondents, 39% reported at least one HP. On average, the worst HPs resolved in less than three weeks. These findings provide valuable insights into the frequency and impact of competition HPs,offering important information for promoters, athletes, coaches, and regulatory bodies to better understand and address the health challenges faced by combat sports athletes.

**Supplementary Information:**

The online version contains supplementary material available at 10.1186/s40798-025-00880-3.

## Key Points

• Among 238 questionnaire responses collected seven days post-competition, two out of every five participants reported at least one health problem (HP).

• Notably, one in ten respondents with a HP were unable to participate in training due to a HP. However, 61.3% of respondents reported no HPs and were able to fully engage in training or did not train for reasons unrelated to a HP. This is particularly reassuring, given the commonly perceived high risk of injuries in mixed martial arts and Muay Thai contests.

• Follow-up data revealed that most self-identified worst HPs resolved within three weeks post-competition, indicating relatively moderate recovery periods for even the most severe HPs.

**Supplementary Information:**

The online version contains supplementary material available at 10.1186/s40798-025-00880-3.

## Background

Outside Olympic combat sports such as boxing, judo, taekwondo, karate, and wrestling, there is growing interest in non-Olympic disciplines such as mixed martial arts (MMA) and Muay Thai (MT). For example, in 2021, the International Olympic Committee (IOC) officially recognised the International Federation of Muay Thai Associations, shortly before MT was included as a medal sport in the 2023 European Games [1]. Similarly, since the inception of the Ultimate Fighting Championship in 1993, both viewership and participation in MMA have grown globally [2, 3]. The characteristics of MMA and MT contests, including duration and rulesets, can vary depending on the organising bodies, competitive levels, and age groups. In Western Australia, the ruleset governing MMA and MT contests are freely available online [4–6]. Typically, contests consist of 3 to 5 rounds, each lasting 2 to 5 min, with a 1-minute break between rounds. In MMA, both striking and grappling techniques are permitted, whereas MT is limited to striking only.

Despite their popularity, participation in MMA [7–12], and MT [13–15], carries inherent risks, with injuries being a frequent consequence. For instance, a systematic review of retrospective studies found that MMA athletes have an injury incidence rate of 246 (males), and 109 (females) per 1000 athlete exposures (AEs) [8]. When adjusted for time at risk, the injury rate for males is 60 per 1000 min of exposure (ME), though comparable data for females is lacking [8]. Regarding MT, a single study reported an injury incidence rate of 55 per 100 AE in a sample of males and females [15]. The most common injuries in MMA are lacerations or abrasions to the head and neck area [7, 8], whereas MT athletes often experience lower extremity injuries like contusions [14, 15], though head injuries are also prevalent [13].

To our knowledge, there have only been five studies examining MT injuries [13–17], and the literature investigating MMA injuries has several shortcomings [7, 8]. Specifically, there is a lack of high-quality studies investigating both injuries and illnesses among MMA and MT athletes. Existing research often underrepresents female participants, lacks detailed MT injury data, and employs inconsistent definitions of reportable injuries, making cross-sport comparisons challenging [2, 7, 8, 13–16, 18]. Additionally, most studies are retrospective and fail to explore the consequences of competition injuries, limiting our understanding of their impact on athletes’ well-being, training, and performance.

To address these gaps, a comprehensive investigation of all health problems (HPs) is needed. This includes capturing the actual time lost from training and adhering to IOC-recommended recording and reporting practices [19, 20]. Even minor injuries can impact an athlete’s training volume and quality [19, 21], and without proper management, they may escalate into more severe conditions requiring extended breaks from sports participation [22]. To ensure comprehensive data collection, this study employs a broad definition of HPs, which includes injuries, illnesses, mental health issues, and pain [20]. This inclusive approach allows for a more holistic understanding of the HPs faced by combat sports athletes, capturing not only physical injuries but also other conditions that may impact their training, performance, and overall well-being.

We aimed to describe the incidence of all competition HPs reported seven days after MMA and MT events, while also examining the consequences of the worst HPs. Follow-up was conducted on the worst HPs to measure their impact, including the number of training days affected and severity scores from the Oslo Sports Trauma Research Centre Questionnaire on Health Problems 2 (OSTRC-H2) [20]. The findings are hoped to inform decision-making, resource allocation, and the development of targeted safety initiatives for combat sports athletes.

## Methods

### Study Design and Ethics

This is a descriptive study of all HPs in MMA and MT competitors. The HPs were collected and reported 7 days after each combat sports event (*n* = 26). Athletes who experienced HPs were followed up weekly until their self-reported worst HP was resolved. Participants were recruited from one or more MMA and MT events held in Western Australia, held between August 2022 and November 2023. The project was developed in collaboration with the staff and board of the Western Australia Combat Sports Commission (WACSC), an organisation responsible for ensuring the health and safety of combat sports athletes in the region. Partial funding for the study was provided by the WACSC. Two of the authors, CD and OB, have close ties to the combat sports community, through training, competition, and coaching. To manage potential conflicts of interest, access to identifiable data provided by the WACSC from non-consenting individuals was limited. To ensure the anonymity of these individuals, the data were separately managed by WACSC staff and author LF. This study (2022-03382-DOHERTY) received approval from the Human Research Ethics Committee of Edith Cowan University. Before accessing the questionnaire, informed consent was obtained from all participants, digitally.

### Participants and Recruitment

To be included in this study, participants had to compete in a sanctioned event regulated by the WACSC, be 18 years of age or older, and possess a sufficient understanding of English to provide informed consent and complete the questionnaires. The WACSC applies legislative requirements prioritising safety, health, and integrity equally across all competitive levels [6]. Therefore, the data are presented as a single group comprising regional, national, and international contests.

The principal investigator (CD) reached out to coaches and event promoters through social media, phone, and email to inform them about the study and request their assistance in recruiting participants. All recruited participants met the athlete registration requirements established by the WACSC. These requirements included submitting a current serology report, testing for human immunodeficiency virus, hepatitis B, and hepatitis C, providing a certification of fitness detailing the participant’s previous weight, current weight, and proposed weight class for competition, and undergoing a pre-contest medical assessment conducted by an assigned physician [6]. Consequently, all participants were deemed healthy before the competition. Athletes who completed the questionnaire in full during a given month were entered into a draw for a $200 gift card for that month. The draw was conducted monthly over 14 months, with only athletes who submitted their responses for that specific month being eligible. Draws were conducted using the online tool https://wheelofnames.com/.

### Definitions

Athlete self-reported HP was defined as *“any condition that you consider to be a reduction in your normal state of full health*,* irrespective of its consequences on your sports participation or performance*,* or whether you have sought medical attention. This may include*,* but is not limited to*,* injury*,* illness*,* pain or mental health”* [20]. Injury was defined as any physical complaint, such as tissue damage or disruption of normal physical function, sustained by a competitor due to sports participation, irrespective of the need for medical attention or time off from activities [19]. Illness refers to complaints or disorders unrelated to injury that affect a body system or manifest as generalised symptoms, and could include any HP affecting physical (e.g., blocked nose), mental (e.g., anxiety), or social well-being [19, 20].

Each HP was classified according to the IOC recommendations for injury by region, area, and type, and for illness by system/region and symptom [19]. If a specific region (e.g., the shoulder) was mentioned multiple times in a questionnaire response due to different types of injuries (e.g., a sprain and a muscle tear), the region itself was counted once. However, all injury types (e.g., sprain and muscle tear) within that region were recorded as separate instances to capture the diversity of injuries affecting the same area.

### Questionnaire and Data Collection Process

All HPs and their consequences were assessed using an online questionnaire, that included the four OSTRC-H2 questions (Supplementary File 1) [20]. The questionnaire was distributed via an anonymised link from Qualtrics (Provo, Utah, USA) 5–7 days after each of the 26 events. All participants received the questionnaire link through their preferred method, such as social media channels (Instagram, Facebook Messenger). If participants reported “full participation without HPs” or “did not train due to other reasons”, the questionnaire ended. However, if “trained with a HP” or “could not participate due to a HP” was selected the respondents proceeded to the injury section. For injuries, a clickable image was provided to select the affected area(s). The participants then specified the type of injury related to the chosen area(s). Participants who reported an injury also responded to a section concerning pain, symptoms, or illnesses, where they could select multiple options. If only one HP was reported, it was considered the worst HP. In instances of multiple HPs, athletes were required to identify the worst HP, as this was the focus of follow-up. They had to explain why it was considered the worst HP, by choosing from a selection of options, or providing their rationale. Consequences of the worst HP were then examined based on the four OSTRC-H2 questions; (1) training participation, (2) training modification, (3) performance, and (4) symptoms/health complaints. Importantly, HP consequences were obtained only for the worst HP. When a respondent selected “full participation without HPs,” all subsequent questions were unnecessary. In this scenario, a total severity score of 0 was given, and the questionnaire was considered complete. When an athlete chooses “could not participate due to a HP,” questions 2 to 4 become redundant [20]. In this case, a total severity score of 100 was assigned. Athletes could also select “did not train for other reasons”, in this case, no score was assigned. Responses to OSTRC-H2 questions 2–4 were scored as follows: 0 = no impact, 8 = mild impact, 17 = moderate impact, and 25 = major/severe impact. The worst HP was deemed substantial if it resulted in moderate or severe reductions in (2) training modification or (3) performance, and it was classified as a time-loss HP if “could not participate due to a HP” was selected [20].

A follow-up assessment was conducted weekly for athletes who reported having a HP until their worst HP resolved or they dropped out. The OSTRC-H2 scoring system, as described above, was used for these assessments. The data collection ended when the worst HP was resolved (e.g., after 21 days). If a participant did not complete a follow-up questionnaire, their data was considered incomplete. To calculate the number of days impacted by the worst HP, we used the date when the athlete reported resuming full participation without any HPs (Supplementary File 1). If athletes did not complete the follow-up, their last questionnaire response date was used. The same method was implemented to calculate the average mean OSTRC-H2 severity score. Non-responders were sent reminder messages if they did not respond to a questionnaire after 2–3 days.

### Exposure-Adjusted Incidence Rates

The WACSC provided the exposure time for bouts. Finish times were only recorded if the bout did not proceed to the final round, for example, in cases of knockout (KO), technical knockout (TKO), or submission. Contests that ended in decisions were calculated by multiplying the number of rounds by the time per round (e.g., 5 rounds x 3 min = 15 min). The recorded finish times were logged on the WACSC’s official contest electronic spreadsheet, which was then uploaded to the departmental record-keeping system. Fight finish times were provided for all questionnaire responses. The official tournament record sheet was not shared by the WACSC to maintain confidentiality. This is because it would involve disclosing individual judges’ names and scoring.

Questionnaire responses with and without HPs were used to calculate the exposure-adjusted incidence rates. Each AE is represented by one contestant in one bout and is considered to reflect one potential exposure. For each sport, the HP, injury, and illness incidence rates per 100 athlete exposures (HPIR_AE_, IIR_AE_, and ILIR_AE_) were calculated:

$$\begin{aligned}&\text{Incidence\:rate\:per}\:100\:\text{AEs}\cr&\quad=\left(\frac{Total\:number\:of\:HPs,\:\:injuries\:or\:illnesses}{Total\:number\:of\:AEs}\right)\cr&\quad\quad\times100\end{aligned}$$

To account for differences in contest exposure time, minutes exposed were used. A minute exposure (ME) is defined as one contestant being exposed for one minute in one bout. The HP, injury, and illness incidence rates per 100 min of exposure (HPIR_ME_, IIR_ME_, and ILIR_ME_) were calculated:

$$\begin{aligned}&\text{Incidence\:rate\:per}\:100\:\text{MEs}\cr&\quad=\left(\frac{Total\:number\:of\:HPs,\:\:injuries\:or\:illnesses}{Toal\:number\:of\:MEs}\right)\cr&\quad\quad\times100\end{aligned}$$

### Data Handling and Analysis

Descriptive analyses were conducted for MMA and MT separately. The findings are presented using various measures, including mean ± standard deviation, median and interquartile range, frequency and proportions, and incidence rates with 95% confidence intervals (CIs), as deemed appropriate. It is worth noting that certain questionnaire responses contained multiple instances of the same HP, categorised as both injury and illness. To avoid duplication, author CD carefully reviewed the data and included only the HPs that were most closely associated with either injury or illness.

The frequency and proportion of injuries are reported by body region (head/neck, upper limb, lower limb, and trunk), area, and type. For illnesses, they are reported by system/region and symptom [19]. The consequences of the worst-reported HPs were calculated using the average mean OSTRC-H2 score, and the median number of days impacted by an HP, based on variable distribution. The median number of days affected was determined by subtracting the HP resolution date, or the date of the last follow-up from the date of the contest. The average mean OSTRC-H2 score was computed by first calculating the cumulative severity score for each worst HP. Then, the individual cumulative scores were divided by the total number of responses for each respondent. Subsequently, the average mean OSTRC-H2 score was calculated and grouped based on the worst HPs. Following the Australian code for responsible research conduct [23], if the cell count for any data was between > 0 and < 4 occurrences, they are presented as “1–3”. Additionally, a range is used (e.g. “4–12” and “13–21”) if the counts of a protected cell could be inferred from adjacent cells. All analyses were conducted using the R Statistical language program (version 4.2.2; R Core Team, 2022).

## Results

**Fig. 1:**
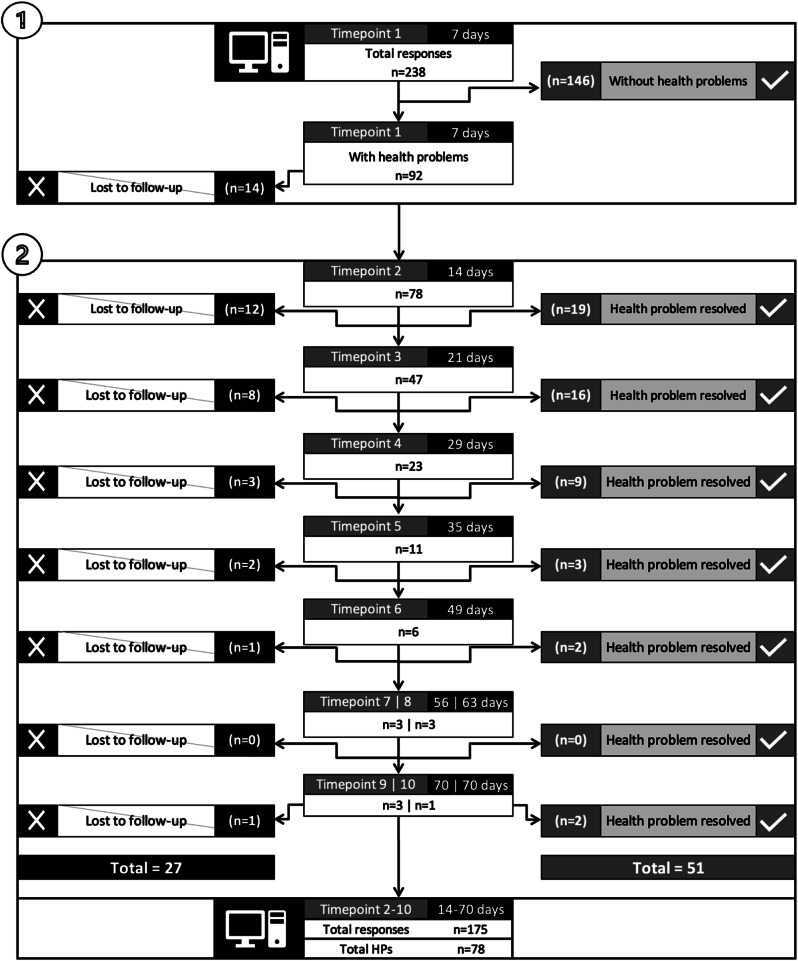
Flow of questionnaire responses. (**1**) Initial contact with athletes 7 days post-competition. (**2**) Follow-up until worst health problem resolution, or participant dropout. The left side shows the loss to follow-up, and the right side shows when athletes reported full training participation without a health problem

**Fig. 2:**
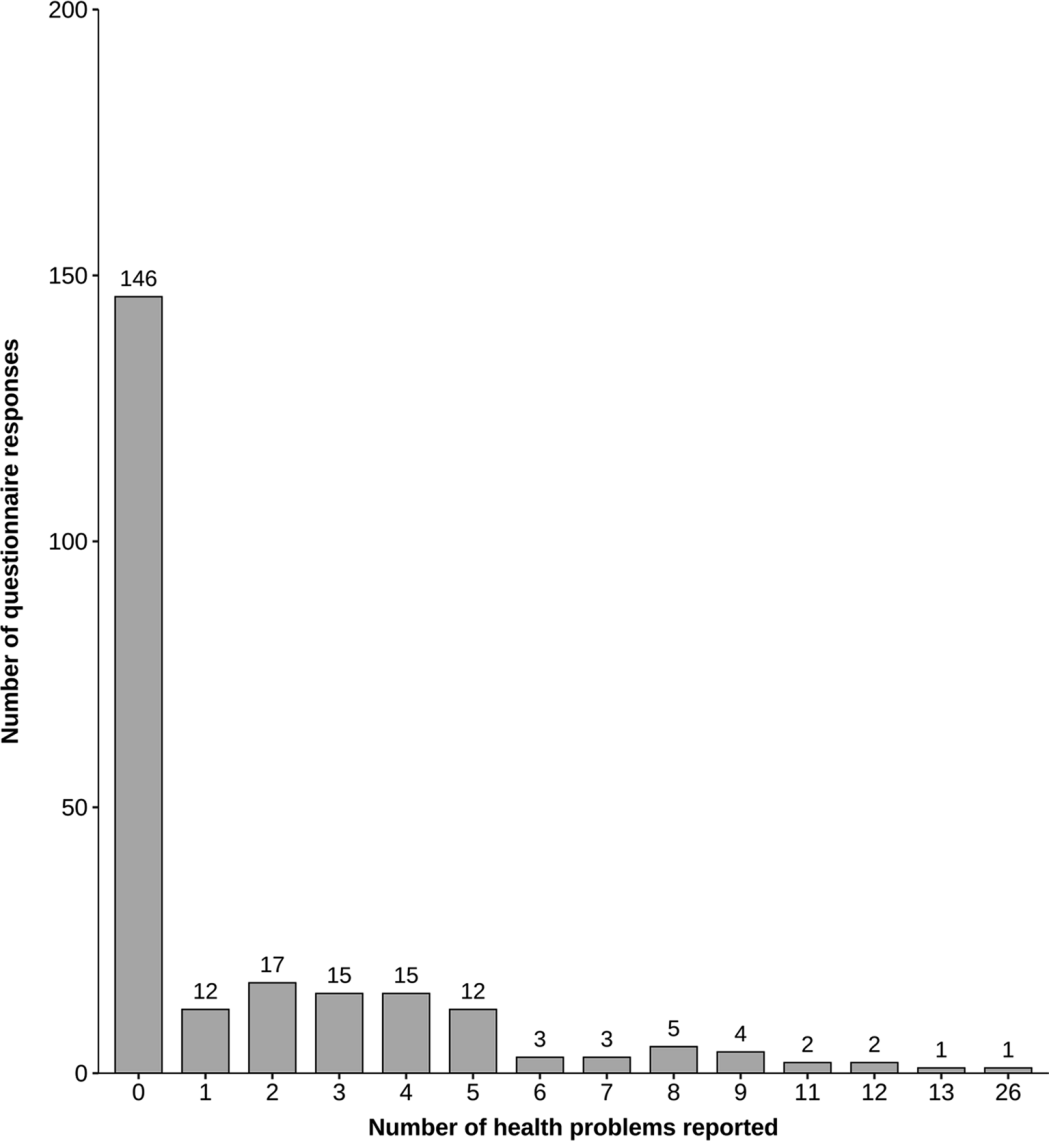
The number of health problems (ranging from 0 to 26) reported by the questionnaire respondents (*n* = 238) at 7 days post-competition

There were a total of 459 potential participants in the 26 events, with 373 males and 86 females. Out of these, 175 competitors (38%) agreed to participate and completed the questionnaire at least once. Among this group, 119 (68%) were MT athletes and 56 (32%) were MMA athletes. Out of the 175 competitors, 126 provided one response, 38 submitted two, and 11 provided three or more responses, resulting in a total of 238 responses over the 26 events at 7 days post-competition (Fig. 1). Six responses contributed a total of 85 HPs, with each reporting between 11 and 26 HPs (Fig. 2). Most contests ended by decision (79%), and there was a similar proportion of respondents who won (54%) or lost (44%) a contest (Table 1). Figure 1 provides a summary of the flow of participants throughout the study, including athletes lost to follow-up and those whose worst HP was resolved.

### Health Problem Incidence and Profile

Out of these 238 questionnaire responses, 146 athletes reported no HPs. Among the 92 responses with HPs, a total of 437 HPs were reported. After review, the final number of HPs was 411, which included 315 injuries and 96 illnesses. The HPIR_ME_ was 25.0 (95% CI: 22.3 to 28.0) for MT, and 20.1 (95% CI: 16.5 to 24.4) for MMA (Table 2). Among the 181 injured regions (Table 3), the lower limb was the most common (61.3%), followed by the upper limb (17.1%) and the head and neck (16%). The proportion of lower limb injuries was 70.4% in MT and 34.8% in MMA. A total of 315 injuries were reported based on tissue and pathology type (Table 4), with the majority being superficial (42.2%) and pain without a specified tissue type (30.5%). Overall, there were 96 illnesses reported across various body systems/regions (Table 5). The most common were nonspecific illnesses (27.1%), followed by upper respiratory (24%) and neurological (22.9%) illnesses.

**Table 1:** Overview of contest characteristics for MMA and MT questionnaire responses. Data are presented as mean ± standard deviation, or count and proportion (%)

Characteristic	MMA	MT	Overall
	*n* = 67	*n* = 171	*n* = 238
**Age (years)**	26 ± 5	27 ± 6	27 ± 6
**Official weight (kg)**	69 ± 9	66 ± 12	67 ± 11
**Response time (days)**	6.2 ± 3.7	6.8 ± 2.6	6.6 ± 2.9
**Reported health problem**			
No	41 (61%)	105 (61%)	146 (61%)
Yes	26 (39%)	66 (39%)	92 (39%)
**Sex**			
Male	63 (94%)	118 (69%)	181 (76%)
Female	4 (6.0%)	53 (31%)	57 (24%)
**Fight outcome**			
Win	33 (49%)	95 (56%)	128 (54%)
Loss	34 (51%)	70 (41%)	104 (44%)
Draw	0	6 (3.5%)	6 (2.5%)
**Type of outcome**			
Decision	37 (55%)	151 (88%)	188 (79%)
KO/TKO	16 (24%)	20 (12%)	36 (15%)
Submission	14 (21%)	0	14 (6%)
**Fight format** **(rounds x minutes)**			
3 × 2	0	114 (67%)	114 (48%)
3 × 3	51 (76%)	20 (12%)	71 (30%)
5 × 2	0	25 (15%)	25 (11%)
3 × 5	12 (18%)	0	12 (5%)
5 × 3	0	12 (7%)	12 (5%)
5 × 5	4 (6%)	0	4 (2%)

Mixed martial arts = MMA, Muay Thai = MT, Technical knockout = TKO, knockout = KO

### OSTRC Score and Days Impacted

Out of the 238 questionnaire responses received 7 days after the competition, 99 athletes (41.6%) reported full participation without any HPs, 47 (19.7%) did not train due to other reasons, 45 (18.9%) had reduced participation because of HPs, 24 (10.1%) were unable to participate due to HPs, 21 (8.8%) had full participation but experienced HPs, and 2 (< 1%) did not answer the OSTRC-H2 questions regarding the consequences of their worst HP.

At 7 days post-competition, the 92 worst HPs were comprised of 91% injuries and 9% illnesses. Injuries mainly affected the lower leg (26%), foot (13%), and head (11%). The most common reasons for athletes’ worst HPs were pain (54%), impaired training (20%), and impaired function (15%). Substantial and time-loss HPs were evaluated based on 90 responses, as two entries were missing for the OSTRC-H2 questions. However, all other data was provided. Among the respondents, 26 reported a substantial HP that impacted their training or performance, while 24 had time-loss HPs (Table 2).

**Table 2:** Overview of questionnaire responses, exposures, health problems, injury, and illness for MMA and MT

Characteristic	MMA			MT			Overall
	n	%		n	%		n
Questionnaire responses	67	28.2%		171	71.8%		238
Athletes	56	32.0%		119	68.0%		175
Questionnaires with HP	26	28.3%		66	71.7%		92
Injury	26	28.3%		66	71.7%		92
Illness	16	29.1%		39	70.9%		55
Athletes with HP	24	29.6%		57	70.4%		81
Injury	24	29.6%		57	70.4%		81
Illness	14	27.5%		37	72.5%		51
Total athlete exposures	67	28.2%		171	71.8%		238
Total minutes exposed	526	30.1%		1221	69.9%		1747
Number of HPs reported	106	25.8%		305	74.2%		411
**Health Problems**							
HP incidence rate per 100 athlete exposures (95% CI)	158.2			178.4			172.7
	(130–191)			(159–200)			(156.4-190.2)
HP incidence rate per 100 min of exposure (95% CI)	20.1			25			23.5
	(16.5–24.4)			(22.3–28)			(21.3–25.9)
**Injury**							
Injury incidence rate per 100 athlete exposures (95% CI)	120.9			136.8			132.4
	(96–150)			(119.9-155.5)			(118.1-147.8)
Injury incidence rate per 100 min of exposure (95% CI)	15.4			19.2			18
	(12.2–19.1)			(16.8–21.8)			(16.1–20.1)
**Illness**							
Illness incidence rate per 100 athlete exposures (95% CI)	37.3			41.5			40.3
	(24.1–55.1)			(32.4–52.4)			(32.7–49.3)
Illness incidence rate per 100 min of exposure (95% CI)	4.8			5.8			5.5
	(3.1-7)			(4.5–7.3)			(4.5–6.7)

Mixed martial arts = MMA, Muay Thai = MT, Confidence Interval = CI, Health problem = HP

**Table 3:** Count and proportion (%) of self-reported injuries by body region and area for MMA and MT questionnaire responses reporting injuries (*n*=92)

Region/area	MMA *n* = 46	MT *n*= 135	Overall *n* = 181
**Head and neck**	**11 (23.9%)**	**18 (13.3%)**	**29 (16%)**
Head	11 (23.9%)	14 (10.4%)	25 (13.8%)
Neck	0	4 (3%)	4 (2.2%)
**Trunk**	**6 (13%)**	**4 (3%)**	**10 (5.5%)**
Chest	1-3	1-3	4 (2.2%)
Abdomen	1-3	1-3	1-3
Lumbosacral	1-3	1-3	1-3
Thoracic spine	1-3	0	1-3
**Upper limb**	**13 (28.3%)**	**18 (13.3%)**	**31 (17.1%)**
Elbow, forearm & wrist	4 (8.7%)	9 (6.7%)	13 (7.2%)
Hand	5 (10.9%)	4 (3%)	9 (5%)
Shoulder	4 (8.7%)	5 (3.7%)	9 (4%)
**Lower limb**	**16 (34.8%)**	**95 (70.4%)**	**111 (61.3%)**
Thigh, lower leg & knee	10 (21.7%)	65 (48.1%)	75 (41.4%)
Foot & ankle	5 (10.9%)	28 (20.7%)	33 (18.2%)
Hip/groin	0	1-3	1-3
Pelvis/buttocks	1-3	0	1-3

Mixed martial arts = MMA, Muay Thai = MT

**Table 4:** Count and proportion (%) of self-reported injuries by tissue and pathology type for MMA and MT questionnaire responses reporting health problems (*n* = 92)

Tissue/pathology type	MMA *n* = 81	MT *n* = 234	Overall *n* = 315
**Pain without tissue type specified**	**22 (27.2%)**	**74 (31.6%)**	**96 (30.5%)**
**Superficial tissues/skin**	**30 (37%)**	**103 (44%)**	**133 (42.**2**%)**
Contusion (superficial)	20 (24.7%)	98 (41.9%)	118 (37.5%)
Laceration	4–12	1–3	8 (2.5%)
Abrasion	4–12	1–3	7 (2.2%)
**Bone**	**12 (14.8%)**	**28 (12%)**	**40 (12.7%)**
Bone contusion	6 (7.4%)	24 (10.3%)	30 (9.5%)
Bone stress injury	1–3	1–3	6 (1.9%)
Fracture	1–3	1–3	4 (1.3%)
**Ligament/joint capsule**	**11 (13.6%)**	**10 (4.3%)**	**21 (6.7%)**
Joint sprain	11 (13.6%)	10 (4.3%)	21 (6.7%)
**Muscle and tendon**	**4–12**	**4–12**	**15 (4.8%)**
Muscle injury	1–3	4–12	13 (4.1%)
Tendon rupture	1–3	1–3	1–3
**Nervous**	**1–3**	**1–3**	**5 (1.6%)**
Brain & spinal cord injury	1–3	1–3	1–3
Peripheral nerve injury	0	1–3	1–3
**Other/unknown/unspecified**	**1–3**	**1–3**	**5 (1.6%)**

Mixed martial arts = MMA, Muay Thai = MT

**Table 5:** Count and proportion (%) of self-reported illness by body system and symptom for MMA and MT questionnaire responses reporting illnesses (*n* = 55)

System/symptom	MMA *n* = 25	MT *n* = 71	Overall *n* = 96
**Non-specific illness**	**8 (32%)**	**18 (25.4%)**	**26 (27.1%)**
**Upper respiratory**	**6 (24%)**	**17 (23.9%)**	**23 (24%)**
Nose issues	1–3	4–12	10 (10.4%)
Throat issues	1–3	4–12	6 (6.3%)
Cough	1–3	1–3	4 (4.2%)
Swollen glands	1–3	1–3	1–3
Covid	1–3	0	1–3
**Neurological**	**5 (20%)**	**17 (23.9%)**	**22 (22.9%)**
Fatigue/malaise	1–3	4–12	11 (11.5%)
Headache	1–3	4–12	9 (9.4%)
Numbness	0	1–3	1–3
**Psychological**	**1–3**	**4–12**	**9 (9.4%)**
Depression/sadness	0	4–12	4–12
Anxiety	1–3	1–3	1–3
Irritability	0	1–3	1–3
**Lower respiratory**	**1–3**	**1–3**	**3 (3.1%)**
Chest pain	1–3	1–3	1–3
Breathing difficulty/tightness	1–3	0	1–3
**Ophthalmological**	**0**	**1–3**	**1–3**
Eye issues	0	1–3	1–3
**Urogenital/Gynaecological**	**1–3**	**0**	**1–3**
Urinary tract/genitalia symptoms	1–3	0	1–3
**Cardiovascular**	**0**	**1–3**	**1–3**
Dizziness	0	1–3	1–3
**Gastrointestinal**	**1–3**	**4–12**	**7 (7.3%)**
Abdominal pain	1–3	1–3	1–3
Diarrhoea	0	1–3	1–3
Constipation	0	1–3	1–3
Nausea	0	1–3	1–3
Vomiting	0	1–3	1–3
**Otological**	**1–3**	**1–3**	**1–3**
Ear issues	1–3	1–3	1–3

Mixed martial arts = MMA, Muay Thai = MT

### Health Problem Follow-up

Among the 81 athletes and 92 responses with an HP at 7 days after the competition, follow-up data were obtained for the worst HPs from 71 out of 81 athletes and 78 out of 92 questionnaire responses (Fig. 1). These data contained 175 responses collected over a period of 14–70 days after the competition. Out of the 51 HPs (15 in MMA and 36 in MT) that had follow-up data until full participation without HPs, 27 dropped out before their HPs were resolved. In Table 6, the median number of days impacted for MMA was 20 (11–27) and for MT was 16 (12–23). The average OSTRC severity score for MT was 39.2 ± 20.6 (*n* = 56) and for MMA was 41.6 ± 20.9 (*n* = 22).

**Table 6:** Overview of the average mean OSTRC-H2 severity score and median days impacted by the worst health problems for MMA (*n* = 22) and MT (*n* = 56). Data from all 10 weekly follow-ups

Worst HP	*n* (%)	MMA Average Score Mean ± SD	MMA Days impacted Median (IQR)	MT Average Score Mean ± SD	MT Days impacted Median (IQR)	Overall Average Score Mean ± SD	Overall Days Impacted Median (IQR)
Abdomen	1–3	54.5	21			54.5	21
Ankle	5 (6.4%)	30.8 ± 6.4	15.5 (13.2–17.8)	46.8 ± 13.2	31 (23.5–32)	40.4 ± 13.2	20 (16–31)
Chest	1–3	64.9	31			64.9	31
Elbow	1–3			33.6	26	33.6	26
Foot	11 (14.1%)	59.5 ± 10.5	44 (33–55)	48.1 ± 20.2	20 (18–26)	50.2 ± 19	21 (18.5–28)
Gastrointestinal	1–3			34	10	34	10
Hand	1–3	23	16.5	41.5	13	29.2	13
Head	7 (9%)	36.3 ± 24	12 (8–34)	43 ± 49.5	18.5 (14.8–22.2)	38.2 ± 28.3	12 (9.5–30)
Hip	1–3			18.7	12	18.7	12
Lower back	1–3	42	13	20.5	10	31.2	11.5
Lower leg	21 (26.9%)	35.7 ± 20.4	50.5 (36.8–64.2)	33.8 ± 16.5	15 (13–22)	34 ± 16.3	16 (13–23)
Neck	1–3			26.5	12	26.5	12
Non- specific illness	1–3			72	23.5	72	23.5
Psychological	1–3			41	14	41	14
Shoulder	5 (6.4%)	36.5 ± 33.9	20 (16–24)	35.8 ± 32.9	16.5 (14.2–18.8)	36.2 ± 29.1	20 (12–21)
Thigh	7 (9%)			42.8 ± 25.7	13 (12–17)	42.8 ± 25.7	13 (12–17)
Upper respiratory	1–3	50	8.5			50	8.5
Wrist	1–3			34.9	26	34.9	26
**Total**	**78**	**41.6 ± 20.9**	**20 (11–27)**	**39.2 ± 20.6**	**16 (12–23)**	**39.9 ± 20.6**	**17 (13–25)**

Mixed martial arts = MMA, Muay Thai = MT, Oslo sports trauma research questionnaire on health problems 2 = OSTRC-H2, interquartile range = IQR, standard deviation = SD. Note: rows with a count of 1–3, only the mean/median value is presented, and the SD or IQR is omitted to maintain data integrity and protect data sensitivity

## Discussion

Both MMA and MT have received limited research attention when it comes to injury and illness complaints that are not solely based on time loss or the need for medical attention. Previous studies have estimated the number of days impacted by injuries but have not performed follow-ups to determine their duration [7]. Therefore, the purpose of this study was to address this gap by describing the incidence of all HPs and conducting follow-ups to determine the duration of the worst HPs. Out of the 238 respondents, 92 reported a total of 411 HPs at 7 days post-competition. Overall, the HPIR_AE_ and HPIR_ME_ were 172.7 and 23.5 per 100 AE. This indicates that there was an average of one self-reported HP per 0.6 AE in MMA and MT contests or one HP for every 4.3 min of exposure. Additionally, injuries accounted for most of the reported HPs, with an overall IIR_AE_ of 121 in MMA and 137 in MT. Despite the high incidence of HPs, most of the worst HPs only impacted training participation and/or performance for an average of 20 days in MMA and 16 in MT.

### Health Problem Incidence

Previous studies have examined the injury incidence rate (IIR) in MMA using various methods, such as analysing injury reports from ringside physicians and reviewing videos [7, 8]. For example, a 2014 meta-analysis found an IIR in MMA of 228.7 (95% CI, 110.4–473.5) per 1000 AEs, though many included studies had poor to moderate methodological quality [7]. Similarly, a 2018 systematic review reported IIRs in MMA ranging from 237 to 286 per 1000 AEs [8]. However, inconsistencies in study designs and variations in recording and reporting practices precluded a meta-analysis. Conversely, Ross et al. (2021) reported a higher IIR of 40 per 100 AEs in professional and amateur MMA competitors [2]. Our findings show an IIR approximately three times higher than that of Ross et al., likely because our study focused on all HPs self-reported by athletes, rather than just injuries documented by ringside physicians. Regarding MT, as far as we are aware, only five studies have examined injuries in this sport [13–17]. One retrospective study of MT athletes reported an IIR of 55 per 100 AEs, which is 2.5 times lower than our findings [15]. Strotmeyer et al. (2016) focused on the most severe single injury [15], whereas we aimed to describe the incidence of all HPs, including less severe complaints such as pain and minor contusions [20]. In contrast, ringside physicians may primarily report more serious injuries that require immediate medical attention, which could explain the discrepancy between our findings and those of previous studies [24]. These methodological differences highlight the need for consistent approaches in combat sports injury research to enable accurate comparisons and tracking of IIRs within and across sports.

In MMA and MT injury research, it is uncommon to report incidence rates normalised by time exposed. However, normalising injury rates to the time an athlete is exposed provides more precise IIRs since contests can end prematurely or go the full scheduled distance, affecting the actual time at risk. Previous MMA studies have reported an IIR per 1000 MEs of 54.3 [7], and 64.9 [25]. Conversely, our study found an overall IIR equivalent to 154 per 1000 MEs, nearly three times higher. Our IIR_ME_ was 15.4 in MMA, while MT had a higher rate of 19.2, which was not evident using the IIR_AE_ measure. Based on the available literature, only one MT study provided time-adjusted measures, reporting an IIR of 2.4 per 100 MEs, whereas our IIR_ME_ was eight times higher [13]. Notably, this study reported only 14 injuries, with just one lower limb injury [13]. These substantial differences in recording and reporting practices underscore the need for a combat sport consensus statement on best practice guidelines, similar to those set by the IOC and the OSTRC-H2 [19, 20].

Illness is seldom studied in MMA and MT. In judo athletes, the ILIR is 3.6 per 1000 AEs for females and 3.0 for males [26]. We found similar ILIR_AE_ and ILIR_ME_ in MMA and MT, although, like injury, it was slightly higher in MT. Based on our findings, 55 of the 92 respondents reported experiencing at least one illness, indicating that athletes often struggle with illnesses during the post-competition period. However, the limited research on this subcategory of HPs suggests that adequate support and targeted solutions are likely insufficient. To our knowledge, this is the first study to publish illness incidence statistics in MMA and MT. Future investigations should aim to include a larger sample of MMA and MT athletes and examine HP incidence, consequences, and risk factors across different competition levels [27], specific sports, and sexes. Such investigations will help identify trends and inform the development of strategies to better support athlete health and well-being.

### Health Problem Profile

In MT, the lower limbs were the most frequently injured body region, with 70.4% of cases affecting this area, consistent with previous research [14, 15]. Specifically, injuries were concentrated in the thigh, lower leg, and knee, accounting for 48.1% of the total injuries. This may be due to the emphasis on leg manoeuvres in the studied MT contests, where leg attacks could have been more prevalent than head or body strikes, leading to a higher incidence of lower limb injuries [15]. Surprisingly, lower limb injuries also predominated among MMA athletes, contrasting with some prior studies that highlighted the head and neck as primary injury sites [8, 28–31]. This disparity might be attributed to the specific characteristics of the MMA contests studied. Like MT, it is conceivable that these contests involved more striking techniques, particularly leg attacks, potentially explaining why the lower limb emerges as the most injured region in MMA. Additionally, the allowance for both striking and grappling techniques in MMA contests might explain the more even distribution of injuries across body regions compared to MT. Recently, our group identified an association between rapid weight changes and both competitive success [32], and injury occurrence [33]. Future research should build upon these findings by investigating whether rapid weight changes are linked to specific injury types, such as head injuries [9, 34–38], and whether potential relationships vary by sex, experience level, and sport.

Overall, we identified similar injury types to those of previous research [7, 8, 14, 15, 18], although considerably fewer lacerations were reported. Interestingly, illnesses represented nearly a quarter of all reported HPs, with nonspecific, upper respiratory, and neurological predominating. Future research should investigate the mechanisms of injury and illness, and whether targeted prevention programs can reduce the reported incidences and affected body regions. Additionally, prospective studies are needed that track athletes over multiple seasons to capture long-term health outcomes beyond competition-related injuries.

### OSTRC Score and Days Impacted

Despite differences in sample sizes and the distinct nature of MMA and MT, notable similarities were observed in the average OSTRC-H2 scores and median days impacted by the worst HPs. For MMA, the median days impacted was 20, while for MT it was 16. The average OSTRC severity score was 39.2 ± 20.6 for MT and 41.6 ± 20.9 for MMA. In MMA, chest injuries had the highest severity scores, whereas lower leg injuries had the longest recovery periods. Conversely, in MT, non-specific illnesses had the highest severity scores, and ankle injuries required the longest recovery periods. Despite these differences in injury nature, it is reassuring that most severe HPs were resolved within three weeks. This quick recovery period is noteworthy, considering the commonly perceived high-risk nature of both sports. It suggests athletes competing in these sports can return to full training participation relatively quickly. To our knowledge, only one previous MT study attempted to examine the consequences of injuries, finding that one-third of participants did not miss any training due to fight-related injuries [15]. Our findings further highlight that, despite the relatively high incidence of HP and injuries, most of the worst HP are typically resolved within three weeks post-competition. These findings have important implications for coaches, medical professionals, and athletes. Understanding common injury patterns and recovery timelines can help develop more effective training regimens, injury prevention programs, and tailored rehabilitation protocols to support athlete performance and long-term health.

### Strengths and Limitations

To the best of our understanding, this study is the first to describe all HPs resulting from MMA and MT events while adhering to IOC-recommended guidelines for recording and reporting [19]. By using event data to determine the actual exposure time, we were able to report precise incidence rates per unit of time exposed. However, these findings should be interpreted within the context of MMA and MT events and not generalised to training or non-competitive settings.

The response rate of this investigation was 39%, which may limit the generalisability of the findings. Athletes who did not participate could differ from respondents in ways that introduce potential response bias. For example, athletes with more severe HPs might have been less likely to respond due to the demands of managing their conditions, or there may have been a reluctance to report sensitive health-related matters. Additionally, 27 respondents who reported HPs at 7 days post-competition dropped out, preventing us from determining the actual number of days they were impacted. This may lead to an underestimation of the overall impact of HPs. Future studies could benefit from strategies to improve response rates and enhance data completeness. Combining ringside physician records with athlete-reported surveys could cross-validate the data, providing a more comprehensive picture of competition-related HPs. Performing follow-up at training facilities for non-responders could further reduce response bias and improve the reliability of the findings.

Another limitation is the lack of data on the participants’ competitive level and the use of protective equipment. Although these variables were not the focus of our research question, their inclusion could provide valuable context for interpreting HPs. Furthermore, some athletes struggled to differentiate between injuries and illnesses, resulting in HPs being reported as both. To avoid double counting, we reviewed these cases and assigned them to one category based on the most relevant information provided. However, we cannot confirm the accuracy of these assignments. To minimise confusion in future studies, one possible approach would be to present illness-related questions only to athletes who specify having an illness, rather than to all injured athletes. Additionally, clarifying whether reported HPs are first-time occurrences or recurring issues would provide a more nuanced understanding of competition-related HPs. Despite these limitations, our study offers new insights into the epidemiology of injuries and illnesses among MMA and MT athletes. It underscores the need for further research to explore a broader range of HPs in these sports and to develop targeted strategies for injury prevention and athlete support.

## Conclusion

In summary, at 7 days post-competition athletes reported one HP for every 0.6 athlete exposures or every 4.3 min of exposure. Despite the high incidence, most of the worst HPs only impacted training participation and/or performance for an average of 17 days. Additionally, illness accounted for almost a quarter of reported HPs, a previously overlooked aspect of post-competition health challenges. These findings provide valuable insights into the frequency and consequences of post-competition HPs, offering important insights for promoters, athletes, coaches, and regulatory bodies to better address the health risks associated with combat sports.

## Electronic Supplementary Material

Below is the link to the electronic supplementary material.

Supplementary Material 1

## Data Availability

The data supporting the findings of this study are not publicly available due to sensitivity reasons. However, interested parties can obtain access to the data by contacting the corresponding author and making a reasonable request.

## Abbreviations

HPs: Health problems
MMA: Mixed martial arts
MT: Muay Thai
IOC: International Olympic committee
AE: Athlete exposure
ME: Minutes of exposure
OSTRC-H2: Oslo Sports Trauma Research Centre Questionnaire on Health Problems 2
WACSC: Western Australia Combat Sports Commission
KO: Knockout
TKO: Technical knockout
HPIR_AE_: Health problem incidence rate per 100 athlete exposures
IIR_AE_: Injury incidence rate per 100 athlete exposures
ILIR_AE_: Illness incidence rate per 100 athlete exposures
HPIR_ME_: Health problem incidence rate per 100 min of exposure
IIR_ME_: Injury incidence rate per 100 min of exposure
ILIR_ME_: Illness incidence rate per 100 min of exposure
CIs: Confidence intervals
