# Supplementary material for: Incidence of Health Problems in Australian Mixed Martial Arts and Muay Thai Competitors: A 14-Month Study of 26 Combat Sports Events
Source: Sports Med Open. 2025 May 28;11:60. doi: 10.1186/s40798-025-00880-3 (PMC12119424; doi:10.1186/s40798-025-00880-3)
Supplement: Supplementary file 1 — Supplementary Material 1 [file 40798_2025_880_MOESM1_ESM.docx]

**Title:** Incidence of Health Problems in Australian Mixed Martial Arts and Muay Thai Competitors: A 14-Month Study of 26 Combat Sports Events.

**Original Investigation**

**Authors:** *Colin S. Doherty, Oliver R. Barley, Lauren V. Fortington

**ORCID ID:**

Colin Doherty: [0000-0002-3589-7490](https://orcid.org/0000-0002-3589-7490)

Oliver Barley: [0000-0002-8543-9818](https://orcid.org/0000-0002-8543-9818)

Lauren Fortington: [0000-0003-2760-9249](https://orcid.org/0000-0003-2760-9249)

**Affiliations:** School of Medical and Health Sciences, Edith Cowan University, Joondalup, WA, Australia.

**Running Head:** Health Problems in Australian Combat Sports Competitors

**Email address and contact details of the corresponding author:**

*Colin Doherty

School of Medical and Health Sciences

Edith Cowan University 270 Joondalup Drive, Joondalup WA 6027, Australia

Phone: +61404068217 Email: [c.doherty@ecu.edu.au](mailto:colindoherty3848@gmail.com)

**Health Problems Questionnaire, including the Oslo Sports Trauma Research Centre Questionnaire on Health Problems 2 (OSTRC-H2)**

Welcome!
 
 This study is investigating health problems as a result of combat sports competition in Western Australia.
 
***”A health problem is any condition that you consider to be a reduction in your normal state of full health, irrespective of its consequences on your sports participation or performance, or whether you have sought medical attention. This may include, but is not limited to, injury, illness, pain or mental health”***

| **WERE YOU INJURED** | | | |
| --- | --- | --- | --- |
| **1** | **Have you participated in any training since the ${Promotion/ChoiceGroup/SelectedChoicesTextEntry} event?** | **Logic** | **Notes** |
|  | Yes | Q2 |  |
|  | No | Q3 |  |
| **2** | **Did you train with a health problem?**  ***”A health problem is any condition that you consider to be a reduction in your normal state of full health, irrespective of its consequences on your sports participation or performance, or whether you have sought medical attention. This may include, but is not limited to, injury, illness, pain or mental health”*** |  |  |
|  | Trained WITH a health problem | Q4 |  |
|  | Full participation WITHOUT health problems | **Finished** |  |
| **3** | **Why have you not participated in training? *”A health problem is any condition that you consider to be a reduction in your normal state of full health, irrespective of its consequences on your sports participation or performance, or whether you have sought medical attention. This may include, but is not limited to, injury, illness, pain or mental health”*** |  |  |
|  | Could not participate due to a health problem | Q4 |  |
|  | Did not participate due to other reasons | **Finished** |  |
| **4** | **What part of the body have you sustained a health problem?** |  |  |
|  | Front of body | Q5 |  |
|  | Back of the body | Q5.1 |  |
|  | Both front and back | Q5 & Q5.1 |  |

Display Q5:

If Q4 = Front of body

Or Q4 = Both front and back

| **5** | **Please click on ALL areas that describes the location of any injuries sustained as a result of your contest at ${Promotion/ChoiceGroup/SelectedChoicesTextEntry}?** |  |  |
| --- | --- | --- | --- |


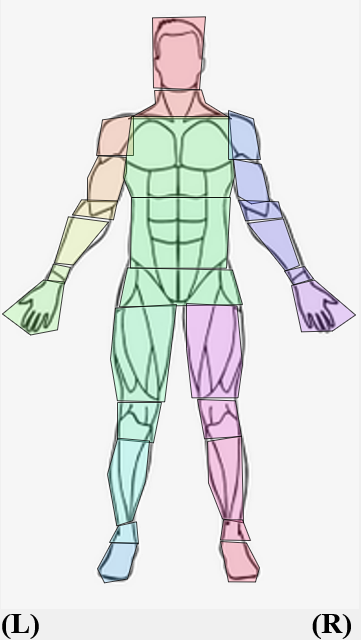


Head

Neck

Shoulder

Upper arm

Forearm

Wrist

Elbow

Hand/fingers

Hip/groin

Thigh

Knee

Lower leg

Ankle

Foot/toes

Chest/ribs

Abdomen

Display Q5.1:

If Q4 = Back of the body

Or Q4= Both front and back


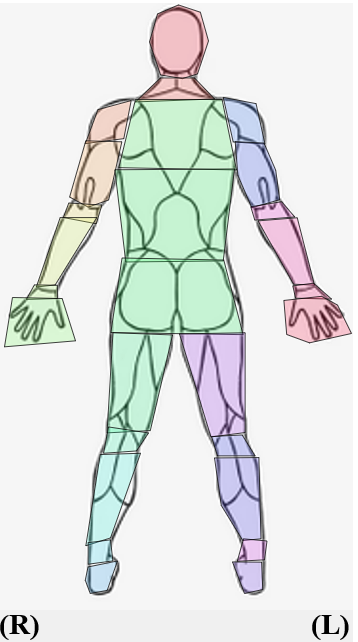


Pelvis/buttocks

Head

Neck

Shoulder

Upper arm

Forearm

Wrist

Elbow

Hand/fingers

Lower back

Upper back

Thigh

Knee

Lower leg

Ankle

Foot/toes

| **5.1** | **Please click on ALL areas that describes the location of any injuries sustained as a result of your contest at ${Promotion/ChoiceGroup/SelectedChoicesTextEntry}?** |  |  |
| --- | --- | --- | --- |

Display Q6:

If Q4 = Front of body

Or Q4= Both front and back

Carry Forward Selected Choices from "FRONT OF BODY"

| **INJURY TYPE: FRONT OF BODY** | | | |
| --- | --- | --- | --- |
| **6** | **Please select the type of injury sustained.** | **Logic** | **Notes** |
|  | Laceration/cut | Q9 | Or Q7 if in Q4 *“Both front and back”* were selected. |
|  | Abrasion/graze/scrape | Q9 |  |
|  | Contusion/bruise | Q9 |  |
|  | Hematoma | Q9 |  |
|  | Bone fracture | Q9 |  |
|  | Bone stress injury | Q9 |  |
|  | Bone contusion/bruise | Q9 |  |
|  | Joint sprain/ligament tear | Q9 |  |
|  | Tendon rupture | Q9 |  |
|  | Muscle strain/tear | Q9 |  |
|  | Nerve injury | Q9 |  |
|  | Concussion | Q9 |  |
|  | Dislocation | Q9 |  |
|  | Dental injury | Q9 |  |
|  | Epistaxis/bloody nose | Q9 |  |
|  | Pain | Q9 |  |
|  | Other | Q8 |  |

Display Q7:

If Q4= Back of the body

Or Q4= Both front and back

Carry Forward Selected Choices from "BACK OF BODY"

| **INJURY TYPE: BACK OF BODY** | | | |
| --- | --- | --- | --- |
| **7** | **Please select the type of injury sustained.** | **Logic** | **Notes** |
|  | Laceration/cut | Q9 |  |
|  | Abrasion/graze/scrape | Q9 |  |
|  | Contusion/bruise | Q9 |  |
|  | Hematoma | Q9 |  |
|  | Bone fracture | Q9 |  |
|  | Bone stress injury | Q9 |  |
|  | Bone contusion/bruise | Q9 |  |
|  | Joint sprain/ligament tear | Q9 |  |
|  | Tendon rupture | Q9 |  |
|  | Muscle strain/tear | Q9 |  |
|  | Nerve injury | Q9 |  |
|  | Concussion | Q9 |  |
|  | Dislocation | Q9 |  |
|  | Dental injury | Q9 |  |
|  | Epistaxis/bloody nose | Q9 |  |
|  | Pain | Q9 |  |
|  | Other | Q8 |  |

Display Q8:

If Q6= Other

Or Q7= Other

| **8** | **You selected “Other” please specify the injury type** | **Logic** | **Notes** |
| --- | --- | --- | --- |
|  | _________________________ | Q9 |  |

| **HEALTH PROBLEM: PAIN/SYMPTOMS/ILLNESS** | | | |
| --- | --- | --- | --- |
| **9** | **Please check ALL the boxes corresponding to any pain/symptoms/illness you have experienced since the ${Promotion/ChoiceGroup/SelectedChoicesTextEntry} event?** | **Logic** | **Notes** |
|  | No pain, symptoms or illness | Q10 |  |
|  | Fever | Q10 |  |
|  | Fatigue/malaise | Q10 |  |
|  | Swollen glands | Q10 |  |
|  | Sore throat | Q10 |  |
|  | Blocked nose/running nose/sneezing | Q10 |  |
|  | Cough | Q10 |  |
|  | Breathing difficulty/tightness | Q10 |  |
|  | Headache | Q10 |  |
|  | Nausea | Q10 |  |
|  | Vomiting | Q10 |  |
|  | Diarrhoea | Q10 |  |
|  | Constipation | Q10 |  |
|  | Fainting | Q10 |  |
|  | Rash/itchiness | Q10 |  |
|  | Irregular pulse/arrhythmia | Q10 |  |
|  | Chest pain/angina | Q10 |  |
|  | Abdominal pain | Q10 |  |
|  | Other pain | Q10 |  |
|  | Numbness/pins and needles | Q10 |  |
|  | Anxiety | Q10 |  |
|  | Depression/sadness | Q10 |  |
|  | Irritability | Q10 |  |
|  | Eye symptoms | Q10 |  |
|  | Ear symptoms | Q10 |  |
|  | Symptoms from urinary tract/genitalia | Q10 |  |
|  | Other (please specify)  ________________ | Q10 | Text entry |

| **WORST HEALTH PROBLEM** | | | |
| --- | --- | --- | --- |
| **10** | **My worst health problem is a _____.** | **Logic** | **Notes** |
|  | Injury (FRONT of body) | Q11 |  |
|  | Injury (BACK of body) | Q12 |  |
|  | Illness | Q13 |  |

Display Q11:

If Q10= Injury (FRONT of body)

Carry Forward Selected Choices from "Front injury type"

| **WORST HEALTH PROBLEM: INJURY (FRONT)** | | | |
| --- | --- | --- | --- |
| **11** | **What is your worst health problem?** | **Logic** | **Notes** |
|  | Head/face | Q14 |  |
|  | Neck | Q14 |  |
|  | Shoulder (L) | Q14 |  |
|  | Upper arm (L) | Q14 |  |
|  | Elbow (L) | Q14 |  |
|  | Forearm (L) | Q14 |  |
|  | Wrist (L) | Q14 |  |
|  | Hand/fingers (L) | Q14 |  |
|  | Chest/ribs | Q14 |  |
|  | Abdomen | Q14 |  |
|  | Hip/groin | Q14 |  |
|  | Thigh (L) | Q14 |  |
|  | Knee (L) | Q14 |  |
|  | Lower leg (L) | Q14 |  |
|  | Ankle (L) | Q14 |  |
|  | Foot/toes (L) | Q14 |  |
|  | Shoulder (R) | Q14 |  |
|  | Upper arm (R) | Q14 |  |
|  | Elbow (R) | Q14 |  |
|  | Forearm (R) | Q14 |  |
|  | Wrist (R) | Q14 |  |
|  | Hand/fingers (R) | Q14 |  |
|  | Thigh (R) | Q14 |  |
|  | Knee (R) | Q14 |  |
|  | Lower leg (R) | Q14 |  |
|  | Ankle (R) | Q14 |  |
|  | Foot/toes (R) | Q14 |  |

Display Q12:

If Q10= Injury (BACK of body)

Carry Forward Selected Choices from "Back injury type"

| **WORST HEALTH PROBLEM: INJURY (BACK)** | | | |
| --- | --- | --- | --- |
| **12** | **What is your worst health problem?** | **Logic** | **Notes** |
|  | Head/face | Q14 |  |
|  | Neck | Q14 |  |
|  | Shoulder (R) | Q14 |  |
|  | Upper arm (R) | Q14 |  |
|  | Elbow (R) | Q14 |  |
|  | Forearm (R) | Q14 |  |
|  | Wrist (R) | Q14 |  |
|  | Hand/fingers (R) | Q14 |  |
|  | Upper back | Q14 |  |
|  | Lower back | Q14 |  |
|  | Pelvis and buttocks | Q14 |  |
|  | Thigh (R) | Q14 |  |
|  | Knee (R) | Q14 |  |
|  | Lower leg (R) | Q14 |  |
|  | Ankle (R) | Q14 |  |
|  | Foot/toes (R) | Q14 |  |
|  | Shoulder (L) | Q14 |  |
|  | Upper arm (L) | Q14 |  |
|  | Knee (L) | Q14 |  |
|  | Lower leg (L) | Q14 |  |
|  | Foot/toes (L) | Q14 |  |
|  | Thigh (L) | Q14 |  |
|  | Ankle (L) | Q14 |  |
|  | Elbow (L) | Q14 |  |
|  | Forearm (L) | Q14 |  |
|  | Wrist (L) | Q14 |  |
|  | Hand/fingers (L) | Q14 |  |

Display Q13:

If Q10 = Illness

Carry Forward Selected Choices - Entered Text from "Illness"

| **WORST HEALTH PROBLEM: ILLNESS** | | | |
| --- | --- | --- | --- |
| **13** | **What is your worst health problem?** | **Logic** | **Notes** |
|  | No pain, symptoms, or illness | Q14 |  |
|  | Fever | Q14 |  |
|  | Fatigue/malaise | Q14 |  |
|  | Swollen glands | Q14 |  |
|  | Sore throat | Q14 |  |
|  | Blocked nose/running nose/sneezing | Q14 |  |
|  | Cough | Q14 |  |
|  | Breathing difficulty/tightness | Q14 |  |
|  | Headache | Q14 |  |
|  | Nausea | Q14 |  |
|  | Vomiting | Q14 |  |
|  | Diarrhoea | Q14 |  |
|  | Constipation | Q14 |  |
|  | Fainting | Q14 |  |
|  | Rash/itchiness | Q14 |  |
|  | Irregular pulse/arrhythmia | Q14 |  |
|  | Chest pain/angina | Q14 |  |
|  | Abdominal pain | Q14 |  |
|  | Other pain | Q14 |  |
|  | Numbness/pins and needles | Q14 |  |
|  | Anxiety | Q14 |  |
|  | Depression/sadness | Q14 |  |
|  | Irritability | Q14 |  |
|  | Eye symptoms | Q14 |  |
|  | Ear symptoms | Q14 |  |
|  | Symptoms from urinary tract/genitalia | Q14 |  |
|  | Other (please specify)  ________________ | Q14 | Text entry |

| **OSTRC-H2 QUESTIONS: CONSEQUENCES OF WORST HEALTH PROBLEM** | | | |
| --- | --- | --- | --- |
| **15** | **Have you had any difficulties participating in normal training due to your worst health problem over the past 7 days?** | **Logic** | **Notes** |
|  | Full participation, but with a health problem | Q16 |  |
|  | Reduced participation due to a health problem | Q16 |  |
| **16** | **To what extent have you modified training due to your worst health problem over the past 7 days?** | **Logic** | **Notes** |
|  | No modification | Q17 |  |
|  | To a minor extent | Q17 |  |
|  | To a moderate extent | Q17 |  |
|  | To a major extent | Q17 |  |
| **17** | **To what extent has your worst health problem affected your performance during the past 7 days?** | **Logic** | **Notes** |
|  | No effect | Q18 |  |
|  | To a minor extent | Q18 |  |
|  | To a moderate extent | Q18 |  |
|  | To a major extent | Q18 |  |
| **18** | **To what extent have you experienced symptoms/health complaints due to your worst health problem during the past 7 days?** | **Logic** | **Notes** |
|  | No symptoms/health complaints | **Finished** |  |
|  | To a mild extent | **Finished** |  |
|  | To a moderate extent | **Finished** |  |
|  | To a severe extent | **Finished** |  |

| **REASON FOR WORST HEALTH PROBLEM SELECTION** | | | |
| --- | --- | --- | --- |
| **14** | **Why is this your worst health problem?** | **Logic** | **Notes** |
|  | Pain | Q15 |  |
|  | Cannot work | Q15 |  |
|  | Cannot train | Q15 |  |
|  | Finance related (i.e., treatment) | Q15 |  |
|  | Impaired function (i.e., cannot walk, sleep issues) | Q15 |  |
|  | Other (please specify) __________________________________________________ | Q15 | Text entry |

Display Q15-Q18:

If Q1 = Yes

**FOLLOW-UP QUESTIONNAIRE**

Welcome back to the study!

| **OSTRC-H2 QUESTIONS: CONSEQUENCES OF WORST HEALTH PROBLEM** | | | |
| --- | --- | --- | --- |
| **1** | **Have you had any difficulties participating in normal training due to your worst health problem over the past 7 days?** | **Logic** | **Notes** |
|  | Full participation without health problems | Q5 |  |
|  | Full participation, but with a health problem | Q2 |  |
|  | Reduced participation due to a health problem | Q2 |  |
|  | Could not participate due to a health problem | **Finished** |  |
| **2** | **To what extent have you modified training due to your worst health problem over the past 7 days?** | **Logic** | **Notes** |
|  | No modification | Q3 |  |
|  | To a minor extent | Q3 |  |
|  | To a moderate extent | Q3 |  |
|  | To a major extent | Q3 |  |
| **3** | **To what extent has your worst health problem affected your performance during the past 7 days?** | **Logic** | **Notes** |
|  | No effect | Q4 |  |
|  | To a minor extent | Q4 |  |
|  | To a moderate extent | Q4 |  |
|  | To a major extent | Q4 |  |
| **4** | **To what extent have you experienced symptoms/health complaints due to your worst health problem during the past 7 days?** | **Logic** | **Notes** |
|  | No symptoms/health complaints | **Finished** |  |
|  | To a mild extent | **Finished** |  |
|  | To a moderate extent | **Finished** |  |
|  | To a severe extent | **Finished** |  |

Please answer the following questions about your WORST health problem.

Display Q5:

If Q1 = Full participation without health problems

| **OSTRC-H2 QUESTIONS: WORST HEALTH PROBLEM RESOLUTION** | | | |
| --- | --- | --- | --- |
| **5** | **What date was full health problem resolution?** | **Logic** |  |
|  | Day ___  Month ___  Year ____ | **Finished** |  |
|  |  |  |  |
